# Identification of an Iron Metabolism-Related lncRNA Signature for Predicting Osteosarcoma Survival and Immune Landscape

**DOI:** 10.3389/fgene.2022.816460

**Published:** 2022-03-11

**Authors:** Shao Hong-bin, Yang Wan-jun, Dong Chen-hui, Yang Xiao-jie, Li Shen-song, Zhou Peng

**Affiliations:** ^1^ Department of Joint Surgery, The 940 Hospital of PLA Joint Logistics Support Force, Lanzhou, China; ^2^ The Second Affiliated Hospital of Xi’an Medical College, Xi’an, China

**Keywords:** osteosarcoma, iron metabolism, lncRNA, immune landscape, signature

## Abstract

**Background:** Long noncoding RNAs (lncRNAs) act as epigenetic regulators in the process of ferroptosis and iron metabolism. This study aimed to identify an iron metabolism-related lncRNA signature to predict osteosarcoma (OS) survival and the immune landscape.

**Methods:** RNA-sequencing data and clinical information were obtained from the TARGET dataset. Univariate Cox regression and LASSO Cox analysis were used to develop an iron metabolism-related lncRNA signature. Consensus clustering analysis was applied to identify subtype-based prognosis-related lncRNAs. CIBERSORT was used to analyze the difference in immune infiltration and the immune microenvironment in the two clusters.

**Results:** We identified 302 iron metabolism-related lncRNAs based on 515 iron metabolism-related genes. The results of consensus clustering showed the differences in immune infiltration and the immune microenvironment in the two clusters. Through univariate Cox regression and LASSO Cox regression analysis, we constructed an iron metabolism-related lncRNA signature that included seven iron metabolism-related lncRNAs. The signature was verified to have good performance in predicting the overall survival, immune-related functions, and immunotherapy response of OS patients between the high- and low-risk groups.

**Conclusion:** We identified an iron metabolism-related lncRNA signature that had good performance in predicting survival outcomes and showing the immune landscape for OS patients. Furthermore, our study will provide valuable information to further develop immunotherapies of OS.

## Introduction

Osteosarcoma (OS) is the most common primary solid malignancy of the bone in children and adolescents, and the overall incidence is ∼4.8 per million worldwide (Lancia et al., 2019; Pingping et al., 2019). The 5-year survival rate of patients with nonmetastatic disease reaches 70–75%; however, the long-term survival rate of metastatic OS is <25% (Anwar et al., 2020). In addition, resistance to chemotherapy or radiation treatments is a common problem for OS treatment (Strauss et al., 2010). Thus, there is an urgent need to identify more efficient targets and novel biomarkers for therapeutic treatment.

Iron is an indispensable element involved in many cellular processes, such as DNA synthesis, ATP production, and oxygen transport (Brown et al., 2020; Forciniti et al., 2020). Iron metabolism is usually divided into distinct processes, including iron acquisition, efflux, storage, and regulation, which is regulated by a set of iron-dependent proteins (Torti & Torti, 2013). Moreover, increasing evidence suggests that dysregulated iron homeostasis and excess iron are crucial risks for cancer development (Morales & Xue, 2021). Ferroptosis is an iron-dependent form of regulated cell death caused by excess levels of reactive oxygen species (ROS) and lipid peroxidation products (Stockwell et al., 2017; Chen et al., 2021). Emerging evidence shows that triggering ferroptosis has anticancer potential for cancer therapy (Liang et al., 2019; Xu et al., 2019).

As a type of noncoding RNA with a length of more than 200 nucleotides, long noncoding RNA (lncRNA) plays important roles in transcriptional regulation and epigenetic gene regulation (Mercer et al., 2009; Kumar & Goyal, 2017). In addition, accumulating evidence shows that lncRNAs act as epigenetic regulators to promote the process of ferroptosis and are involved in iron metabolism (Wu et al., 2020). For example, the report showed that lncRNA RP11-89 promoted tumorigenesis of bladder cancer and inhibited ferroptosis through PROM2-activated iron export (Luo et al., 2021). Another study suggested that lncRNA MT1DP improved the sensitivity of erastin-induced ferroptosis and increased the intracellular ferrous iron concentration in non-small-cell lung cancer (NSCLC) (Gai et al., 2020). However, there is still a lack of reports on the iron metabolism-related lncRNAs in osteosarcoma. Hence, there is an urgent need to develop novel iron metabolism-related lncRNA signatures for the diagnosis and prognosis of OS.

**FIGURE 1:**
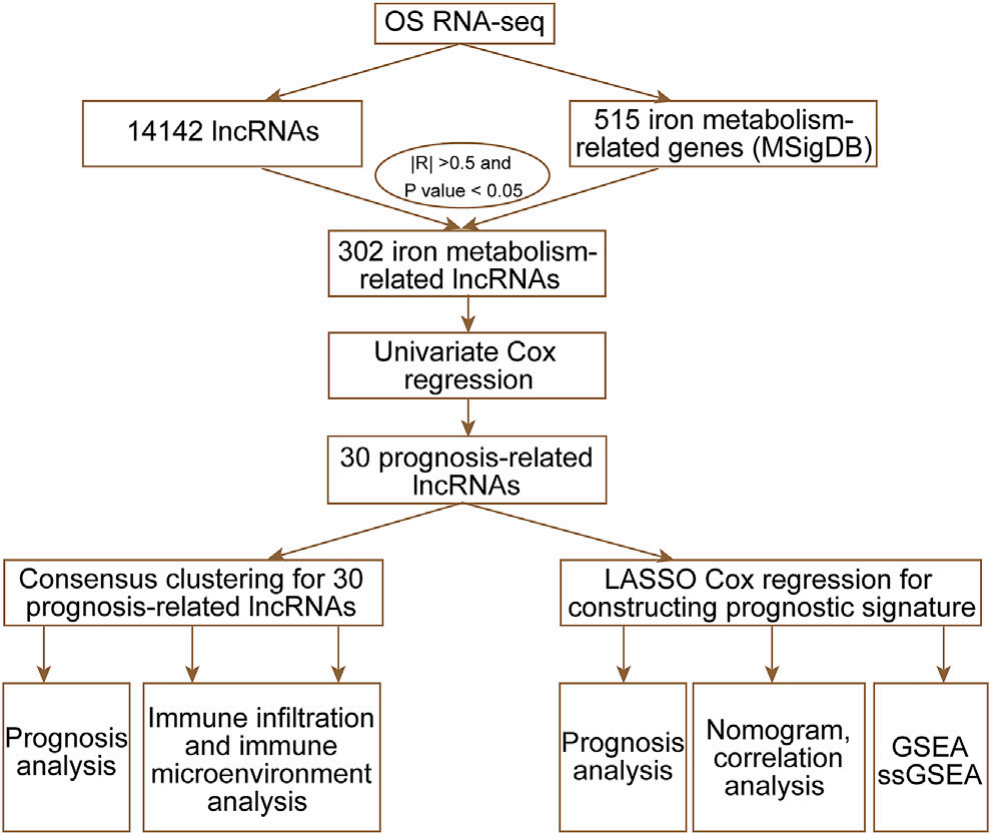
Workflow of the current study.

In this study, we downloaded RNA-sequencing and clinical data from Therapeutically Applicable Research to Generate Effective Treatments (TARGET) and obtained iron metabolism-related gene sets from the Molecular Signatures database v7.4 (MSigDB). Through univariate Cox regression and consensus clustering analysis, we estimated immune infiltration and clinical features in two prognosis-related lncRNAs and molecular subtypes. Then, we applied least absolute shrinkage and selection operator (LASSO) regression analysis to construct an iron metabolism-related lncRNA signature. Finally, we established nomograms to predict prognosis and developed a treatment strategy for OS patients. The workflow of the study is shown in Figure 1.

## Methods

### Data Collection and Preprocessing

RNA-seq data and clinical information of OS patients were downloaded from Therapeutically Applicable Research to Generate Effective Treatments (TARGET; https://ocg.cancer.gov/programs/target). Finally, we obtained 84 patients’ RNA-seq data and clinical information. From the Molecular Signatures database v7.4 (MSigDB) (Liberzon et al., 2015), we extracted 515 iron metabolism-related genes from 15 iron metabolism-related gene sets.

### Identification of Iron Metabolism-Related lncRNAs and Prognosis-Related lncRNAs

The Perl programming language was used to obtain the expression matrix of iron metabolism-related genes and lncRNAs from the TARGET dataset. The 302 iron metabolism-related lncRNAs were selected based on the criteria that the absolute value of the correlation coefficient was greater than 0.5 (|R|>0.5) and the *p*-value was less than 0.05 (*p* < 0.05). Through univariate Cox regression analysis, we identified 30 iron metabolism-related lncRNAs whose expression levels were significantly associated (*p* < 0.05) with the overall survival of OS patients.

### Consensus Clustering

ConsensusCluster usually generates a consensus matrix heatmap and a log of selected features that distinguish each pair of clusters. Based on 30 prognosis-related lncRNAs, we applied the “ConsensusClusterPlus” software package (Wilkerson & Hayes, 2010) to perform consensus clustering analysis. Meanwhile, we also obtained unbiased and unsupervised outcomes for approximately 84 OS patients. According to two different regulation patterns, we visualized the expression of 30 prognosis-related lncRNAs by using the R package “pheatmap” (Kolde, 2015).

### Estimation of the Immune Microenvironment and Immune Infiltration

We estimated the parameters of the immune microenvironment (stromal score, immune score, and ESTIMATE score) through the “estimate” software package (Yoshihara et al., 2013) in Cluster 1 and Cluster 2. Moreover, we applied CIBERSORT (Newman et al., 2019) to analyze the expression matrix of immune cell subtypes between Cluster 1 and Cluster 2.

### Construction and Evaluation of an Iron Metabolism-Related lncRNA Signature

LASSO Cox analysis was used to obtain an optimal iron metabolism-related lncRNA signature by using the R package “glmnet” (Friedman et al., 2010). Based on the best lambda value and coefficients, the risk score of each OS case could be obtained by the following algorithm:

$$\mathrm{Risk}\;\mathrm{Score}=\sum_{i\mathrm{=1}}^{n}\mathrm{Coe}f_{i}*E_{i},$$

where n, Coef_*i*_, and E_*i*_ represent the number of signature genes, coefficient of a gene, and expression of a gene, respectively. The Kaplan–Meier survival curve was used to assess the overall survival of the high- and low-risk groups by using the R package “survival” (Lorent et al., 2014). Principal component analysis (PCA) was used to evaluate distribution patterns between high- and low-risk groups based on an iron metabolism-related lncRNA signature through the “ggplot2” software package (Gómez-Rubio, 2017). Moreover, the receiver-operating characteristic (ROC) curves were applied to evaluate the diagnostic efficacy of each clinicopathological characteristic and the prognostic signature through the “survivalROC” software package (Heagerty et al., 2000). Finally, we applied multivariate Cox regression analyses to evaluate the independent prognostic value between the risk score and other clinical variables, such as age, sex, and the prognosis stage by using the “forestplot” package (Gordon & Lumley, 2016).

### Nomogram Construction

A nomogram was constructed to analyze the probable 1-, 3-, and 5-year overall survival of the OS patients by using the R package “rms” (Bandos et al., 2009). 3- and 5-year calibration curve analyses were used to evaluate the suitability of our nomogram.

### Gene Set Enrichment Analysis

The “ConsensusClusterPlus” software package was used for enrichment. GSEA (http://www.broadinstitute.org/gsea/index.jsp) was used to evaluate the differentially enriched genes between the high- and low-risk groups. “c2. cp. kegg.v7.2. symbols. gmt” and “c5. go. v7.4. symbols. gmt” were used as the reference gene sets. The criterion of statistically significant enrichment was | NES | > 1 and *p*-value < 0.05.

### Statistical Analysis

R software (version 4.0.2; https://cran.r-project.org/bin/windows/base/) and various R packages were used for all statistical analyses and visualization in this study. Perl (version 5.8.3; https://www.perl.org/get.html) was applied to integrate RNA-seq data and clinical information for screening prognosis-related genes. The criterion of statistical significance was *p*-value < 0.05.

## Results

### Identification of Iron Metabolism-Related lncRNAs and Prognosis-Related lncRNAs

**FIGURE 2:**
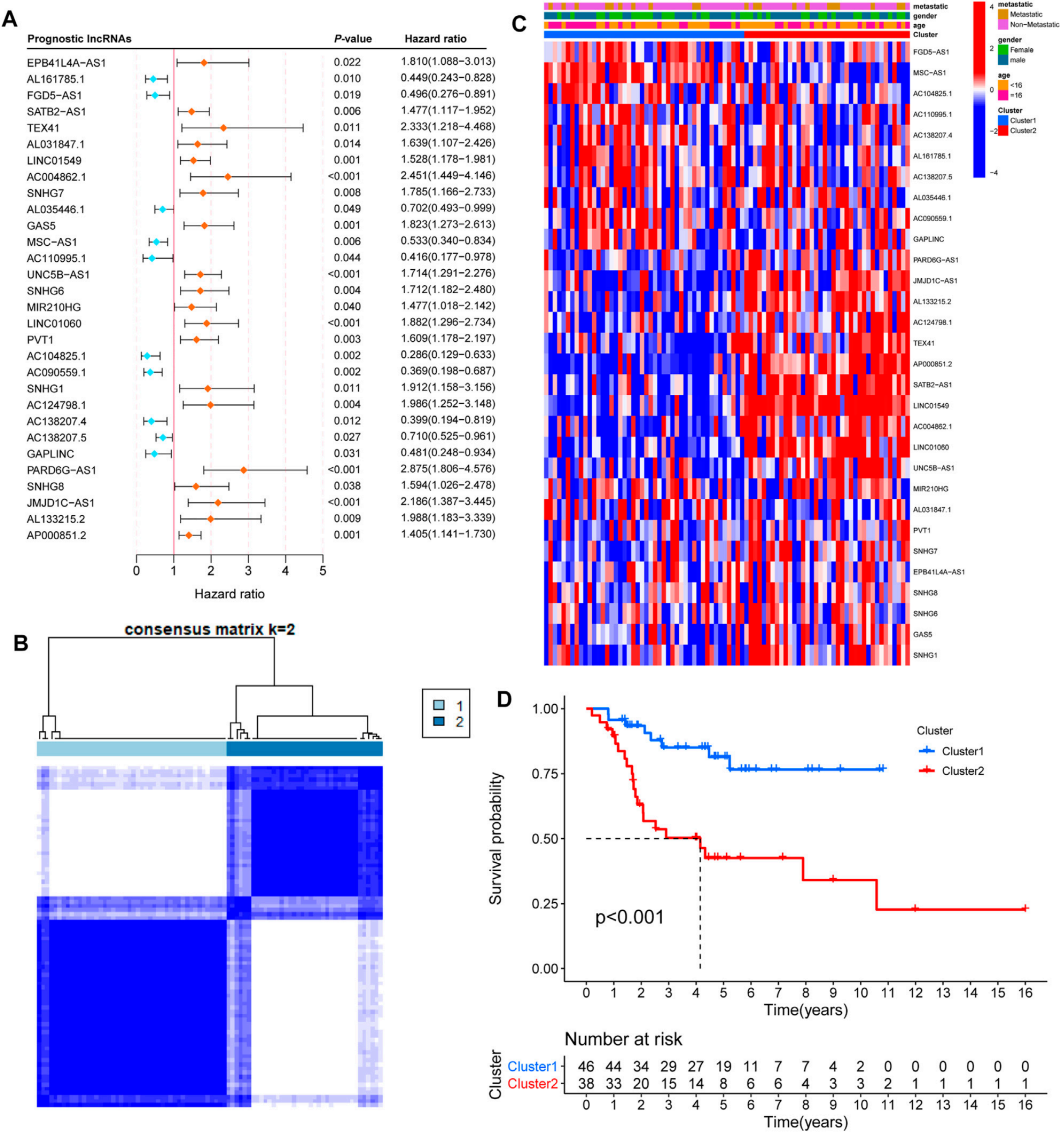
Consensus clustering by prognosis-related lncRNAs. **(A)** Univariate Cox regression analysis showed that 30 iron metabolism-related lncRNAs significantly correlated with the overall survival of OS patients. Orange and bluish dots represent the point estimation of the hazard ratio. **(B)** The consensus score matrix of 84 samples when k = 2. **(C)** Heatmap with clinical information for 30 iron metabolism-related lncRNAs. **(D)** Kaplan–Meier curves of patients in two clusters.

To identify iron metabolism-related lncRNAs, we first extracted 515 iron metabolism-related genes from 15 iron metabolism-related gene sets (Supplementary Table S1). Moreover, we obtained an expression matrix of 14,142 lncRNAs from RNA-sequencing data. According to the criteria |R| >0.5 and *p*-value <0.05, 302 lncRNAs were regarded as iron metabolism-related lncRNAs. Then, we combined the expression of 302 iron metabolism-related lncRNAs and clinical information to screen prognosis-related lncRNAs (Table 1). Univariate Cox regression analysis showed that 30 iron metabolism-related lncRNAs significantly correlated with the overall survival of OS patients (Figure 2A). Meanwhile, we found that the expression levels of 10 prognosis-related lncRNAs were negatively related to the survival rate, while 20 other lncRNA prognosis-related lncRNAs were positively associated with the survival rate.

**TABLE 1:** Clinical features of all patients.

Feature	Group	TARGET dataset (n = 84)
		Number of patients (%)
Age	<16	48 (57.1)
	≥16	36 (42.9)
Gender	Female	36 (42.9)
	Male	48 (57.1)
Metastatic	Metastatic	21 (25.0)
	Non-metastatic	63 (75.0)
Histologic response	Stage 1/2	18 (21.4)
	Stage 3/4	16 (19.0)
Vital status	Alive	55 (65.5)
	Dead	29 (34.5)

### Consensus Clustering by Prognosis-Related lncRNAs

To gain insight into the function of iron metabolism-related lncRNAs, we applied unsupervised consensus analysis to the expression levels of 30 prognosis-related lncRNAs. The results showed that k = 2 seemed to be a more accurate and stable clustering. Meanwhile, Cluster 1 and Cluster 2 included 46 and 38 samples, respectively (Figure 2B). The results of k = 3–9 are shown in Supplementary Figure S1. Subsequently, we explored the difference between the two clusters. First, the heatmap of 30 prognosis-related lncRNAs showed differential expression in the two clusters (Figure 2C). Based on gender, age, and stage-specific characteristics, differential expression of 30 prognosis-related lncRNAs is shown in Supplementary Figures S2A–C. Additionally, the Kaplan–Meier survival curve analysis suggested that the overall survival of Cluster 2 patients was significantly shorter than that of Cluster 1 patients (Figure 2D).

### Immune Infiltration and the Immune Microenvironment in Two Prognosis-Related lncRNA Clusters

**FIGURE 3:**
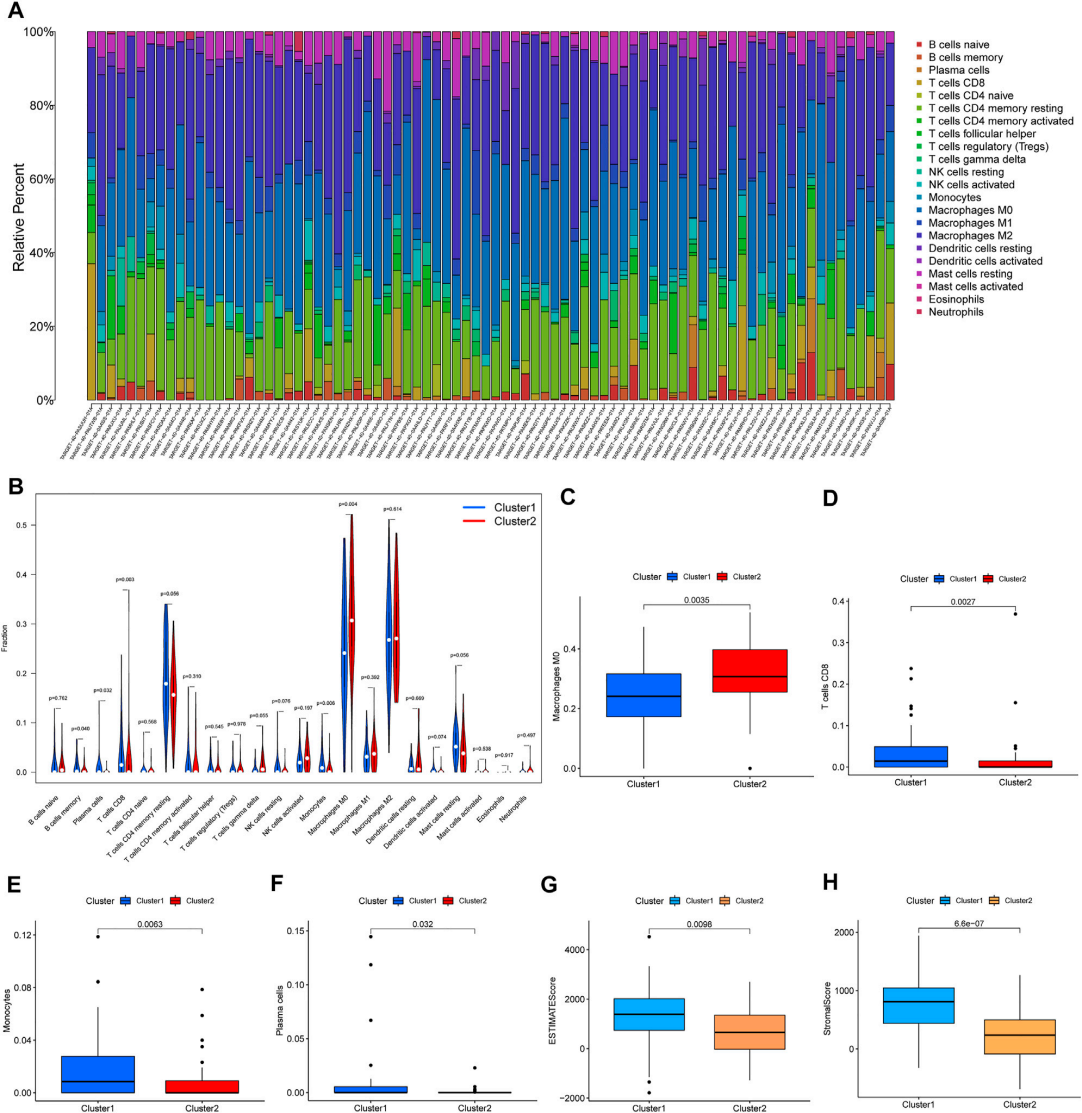
Immune infiltration and the immune microenvironment in the two clusters. **(A)** The proportions of 22 types of immune cells in 84 samples. **(B)** The significant analysis for the same type of immune cell proportions in the two clusters. **(C–F)** The results for proportions of macrophages, CD8 T cells, monocytes, and plasma cells. **(G,H)** ESTIMATE score and stromal score in two clusters.

Furthermore, we analyzed the difference in immune infiltration and the immune microenvironment in the two prognosis-related lncRNA clusters. The percentage of 22 immune cells is shown in Figure 3A. We found that M0 macrophages presented the largest proportion and eosinophils accounted for a small scale. A violin plot was used to further explore the difference in 22 immune cell types in the two clusters (Figure 3B). The results of immune infiltration showed that Cluster 1 was observably enriched with B-cell memory, plasma cells, CD8 T cells, and monocytes (Figures 3D–F). Only M0 macrophages were enriched in Cluster 2 (Figure 3C). In addition, enrichment of M2 macrophages was not significant between Cluster 1 and Cluster 2 (Supplementary Figure S2D). The difference in the immune microenvironment was estimated by the analysis of the stromal score, immune score, and ESTIMATE score. The results of the stromal score and ESTIMATE score showed that there was a significantly higher score in Cluster 1 than in Cluster 2 (Figures 3G,H), and the immune score did not present a significant result. Taken together, these results confirmed that Cluster 1 and Cluster 2 had different immune phenotypes.

### Construction of an Iron Metabolism-Related lncRNA Signature

**FIGURE 4:**
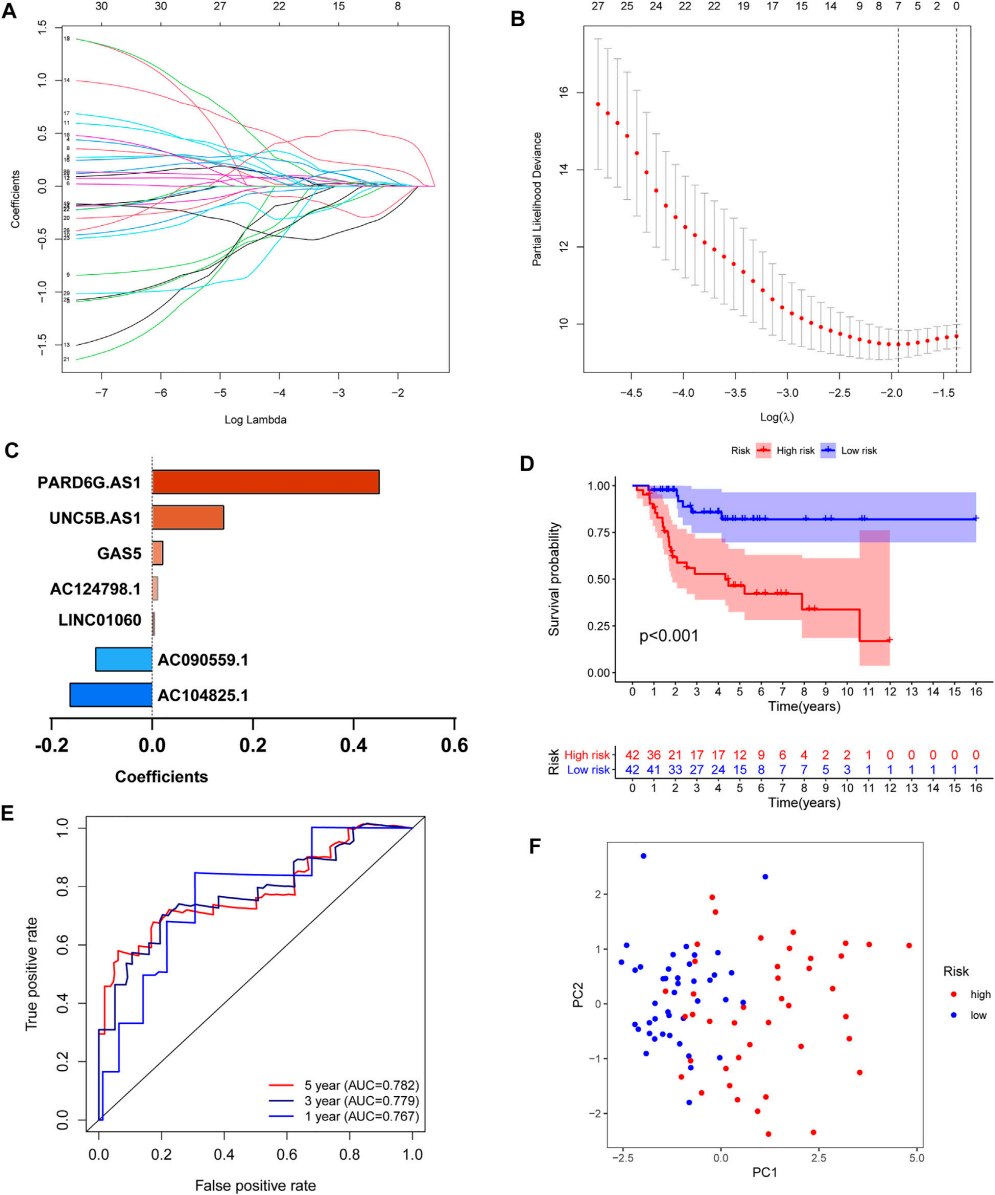
Construction of an iron metabolism-related lncRNA signature. **(A,B)** LASSO Cox regression analysis showed that seven iron metabolism-related lncRNAs were good candidates for constructing the prognostic signature (λ = 0.0125, obtained seven nonzero coefficients). **(C)** Coefficients of the seven iron metabolism-related lncRNAs. **(D)** Kaplan–Meier curves of patients in the high- and low-risk groups. **(E)** ROC curve to evaluate the 1/3/5-year prediction efficiency of the iron metabolism-related lncRNA signature. **(F)** Principal component analysis (PCA) based on the identified iron metabolism-related lncRNA signature.

To further develop the prognostic signature, we performed LASSO Cox regression analysis for 30 prognosis-related lncRNAs. The analysis showed that seven of the thirty iron metabolism-related lncRNAs were good candidates for constructing the prognostic signature. When lambda was minimum (0.1445), the number of nonzero coefficients was seven in the model at this time (Figures 4A,B). Meanwhile, the coefficients of five lncRNAs (PARD6G.AS1, GAS5, UNC5B.AS1, LINC01060, and AC124798.1) were positive, while AC090559.1 and AC104825.1 were negative (Figure 4C). Based on the iron metabolism-related lncRNA signature, OS patients were divided into high-risk (*n* = 42) and low-risk (*n* = 42) groups. Then, the Kaplan–Meier survival curve analysis, receiver-operating characteristic (ROC) analysis, and principal component analysis (PCA) were used to examine the prognostic value of iron metabolism-related lncRNA signatures. The Kaplan–Meier survival curve analysis suggested that the overall survival of OS patients with high-risk scores was significantly shorter than that of patients with low-risk scores (Figure 4D). ROC analysis presented a good prediction efficiency of the iron metabolism-related lncRNA signature (1-year AUC = 0.767, 3-year AUC = 0.779, and 5-year AUC = 0.782; Figure 4E). Finally, PCA revealed two relatively different distribution patterns between high- and low-risk groups (Figure 4F).

### Independent Prognostic Value and the Predictive Prognostic Ability of the Risk Score Model

**FIGURE 5:**
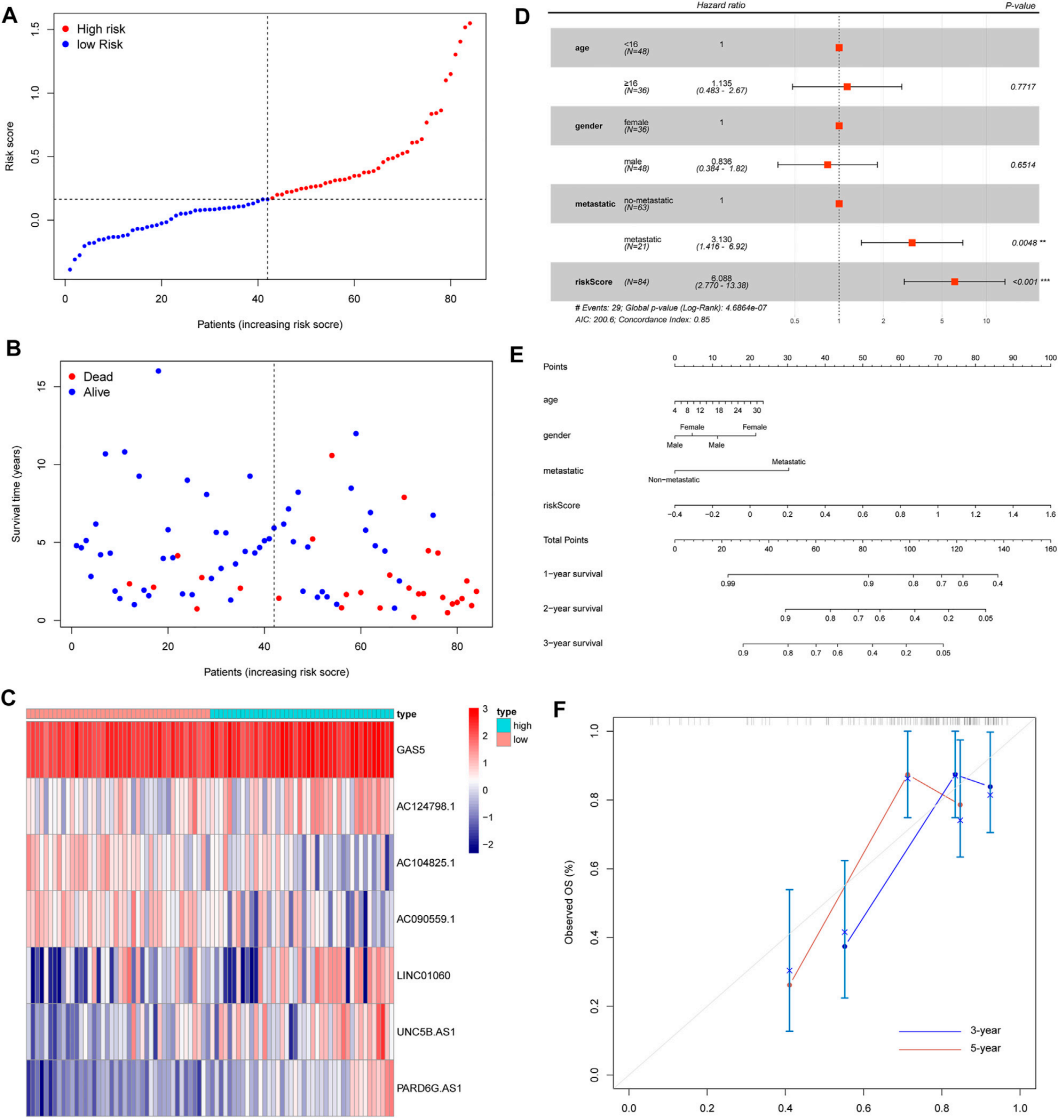
Evaluation of an iron metabolism-related lncRNA signature. **(A)** Risk score distribution. **(B)** Survival time of OS patients. **(C)** Heatmap of seven iron metabolism-related lncRNAs. **(D)** Multivariate Cox analyses to evaluate whether the risk score and clinical features were independent prognostic indicators for the overall survival of OS patients. **(E)** Nomogram for predicting 1/3/5-year survival rates of OS patients. **(F)** Calibration curve of the nomogram for 3/5-year survival rates.

Based on the iron metabolism-related lncRNA signature, we visualized the risk score distribution and survival status of every sample (Figures 5A,B). We also generated a heatmap of the seven iron metabolism-related lncRNAs (Figure 5C). Moreover, the respective Kaplan–Meier survival curves of seven iron metabolism-related lncRNAs showed that they were significantly correlated with the overall survival of patients (Supplementary Figures S3A–G). Then, we applied multivariate Cox analyses to evaluate the prognostic value of the risk score model. The results suggested that tumor metastasis and the risk score were significantly correlated with the overall survival of patients (Figure 5D). Compared with the iron metabolism-related lncRNA signature, ROC analysis of clinicopathological factors did not have an advantage in predicting prognosis (Supplementary Figure S3H). Additionally, we constructed a nomogram and calibration curve to accurately estimate the 1-, 3-, and 5-year survival probabilities based on the risk score and other clinicopathological factors, including age, gender, and the prognosis stage (Figures 5E,F). Overall, the above data verified that the risk score model had good performance in predicting the overall survival of OS patients.

### Different Immune-Related Functions Between the High- and Low-Risk Groups

**FIGURE 6:**
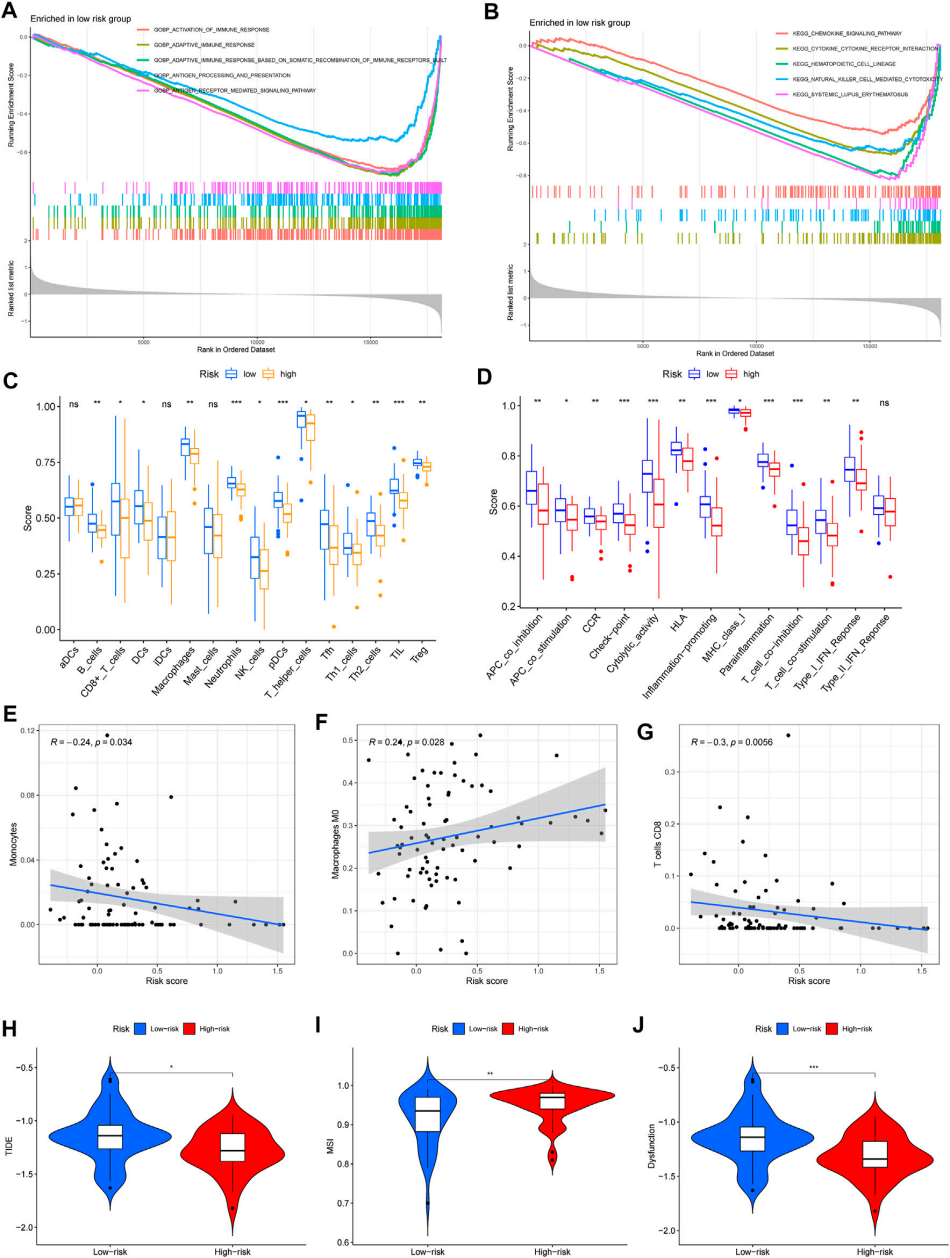
Immune-related functions and immunotherapy response between the high- and low-risk groups. **(A,B)** Significantly enriched GO terms and KEGG pathways in low-risk groups. **(C,D)** Scores of immune cells and immune-related pathways in low- and high-risk groups. **(E–G)** The significant correlation between immune infiltration and risk scores, including monocytes, M0 macrophages, and CD8 T cells. **(H–J)** Analysis of TIDE, microsatellite instability (MSI), and dysfunction (^∗^ *p* < 0.05; ^∗∗^ *p* < 0.01; and ^∗∗∗^ *p* < 0.001).

Based on the iron metabolism-related lncRNA signature, we subsequently carried out GSEA to examine the potential biological processes involved. Interestingly, the GSEA results showed that the most significant GO terms and KEGG pathways were mainly enriched in low-risk groups, and there were no significant terms or pathways enriched in the high-risk groups (Figures 6A,B). Then, we applied single-sample gene set enrichment analysis (ssGSEA) to compare scores of immune cells and immune-related pathways between high- and low-risk groups. The ssGSEA results confirmed that the scores of immune cells and immune-related pathways in low-risk groups were significantly higher than those in high-risk groups (Figures 6C,D). Based on the immune phenotypes, the GSEA score and Kaplan–Meier survival curve analysis, we speculated that the high-risk groups and Cluster 2 presented an immune-excluded phenotype. In addition, low-risk groups and Cluster 1 showed an immune-inflamed phenotype and presented a better survival (Chen & Mellman, 2017).

### Immunotherapeutic Response Based on the Risk Score Model

Except for the above immune cell and immune-related pathway scores, we also calculated the correlation between immune infiltration and the risk score. The results confirmed that monocytes and CD8T cells were negatively related to the risk score, but M0 macrophages were positively associated with the risk score (Figures 6E–G). Then, we used the tumor immune dysfunction and exclusion (TIDE) algorithm to evaluate the immunotherapy response between the high- and low-risk groups. Consistently, we found that TIDE, microsatellite instability (MSI), and dysfunction were significantly higher in low-risk groups (Figures 6H–J). These results indicated that high-risk groups had a good response to immunotherapy.

## Discussion

Recently, increasing evidence has indicated that iron metabolism is a critical factor that promotes the carcinogenesis of OS (Lv et al., 2020). Meanwhile, many reports revealed that improving the process of iron metabolism, such as iron deprivation, might be an effective strategy for OS treatment (Li et al., 2016; Ni et al., 2020). In our study, we identified 30 iron metabolism-related lncRNAs that were related to OS prognosis. Meanwhile, the results of consensus clustering of 30 prognosis-related lncRNAs showed the differences in immune infiltration and the immune microenvironment in the two clusters. Through univariate Cox regression and LASSO Cox regression analysis, we constructed an iron metabolism-related lncRNA signature including seven iron metabolism-related lncRNAs. The signature was verified to have good performance in predicting the overall survival, immune-related functions, and immunotherapy response of OS patients between the high- and low-risk groups.

As important epigenetic regulators, lncRNAs play a crucial role in the processes of iron metabolism (Wu et al., 2020). Moreover, accumulating evidence confirmed that lncRNAs are involved in the regulation of ferroptosis and may serve as effective targets for cancer treatment (Jiang et al., 2021; Xie & Guo, 2021). In this study, we developed an iron metabolism-related lncRNA signature including seven iron metabolism-related lncRNAs. In particular, lncRNA GAS5, UNC5B-AS1, PARD6G-AS1, and LINC01060 have been widely reported in cancer and other diseases. For example, it was proven that lncRNA GAS5 was associated with OS progression by regulating OS cell proliferation, migration, and invasion (Ye et al., 2017; Yao et al., 2020; Wang et al., 2021). Moreover, a report showed that lncRNA GAS5 was associated with ovarian cancer progression by regulating histone H3 at lysine 27 (H3K27me3) (Wang et al., 2020). However, except for the reports that lncRNA GAS5 correlated with OS progression, there was no evidence that other iron metabolism-related lncRNAs were involved in OS progression *in vitro* and *in vivo*. Therefore, OS treatment will be necessary to explore the molecular biological functions of the seven iron metabolism-related lncRNAs in the processes of iron metabolism and ferroptosis.

Iron metabolism is closely related to the immune system, including regulating immune cell proliferation, differentiation, and interfering with antimicrobial immune effectors (Ganz & Nemeth, 2015; Cronin et al., 2019; Haschka et al., 2021). Furthermore, imbalances in iron metabolism, including iron overload and iron deficiency, have an important effect on immune function (Porto & De Sousa, 2007; Cassat & Skaar, 2013; Nairz & Weiss, 2020). In our study, we found that many kinds of immune infiltration were enriched in Cluster 1. Meanwhile, the stromal score and ESTIMATE score were significantly higher in Cluster 1 than in Cluster 2. Taken together, we divided 84 samples into two clusters based on 30 iron metabolism-related lncRNAs and evaluated the different immune infiltrations and microenvironments. Therefore, focusing on the regulation of iron metabolism-related lncRNAs will be a novel strategy to change iron metabolism and improve immune functions.

To date, the tumor microenvironment is regarded as having a critical role in cancer development and treatment, and has become one of the most important factors affecting immunotherapy (Smyth et al., 2016; Kaymak et al., 2021). Meanwhile, strategies focusing on immunotherapy have been an increasingly attractive treatment option for OS patients (Dyson et al., 2019; Chen et al., 2021). In our study, the GSEA results confirmed that significant GO terms and KEGG pathways were mainly enriched in low-risk groups and were associated with immune pathways. Additionally, it was also found that the high-risk group presented an immune-excluded phenotype and the low-risk group showed an immune-inflamed phenotype. For the poorer prognosis of high-risk patients, we speculated that higher immunosuppression and lower immunoreactivity should have been the causes. Interestingly, we found that the high-risk groups well responded to immunotherapy through the TIDE algorithm. Therefore, this evidence seems to provide a theoretical basis for applying immunotherapy to OS patients in the future.

Although the iron metabolism-related lncRNA signature showed the ability to potentially predict prognosis, there are several limitations to our study. Except for RNA-sequence data and clinical information from the TARGET, there is a need to evaluate the value of the risk score model in the other testing dataset. Moreover, the risk score model showed a certain predictive ability for OS prognosis, and it is still necessary to verify this prediction in large cohorts.

In conclusion, we identified an iron metabolism-related lncRNA signature that had good performance in predicting survival outcomes and showed the immune landscape for OS patients. Furthermore, our study will provide valuable information to further develop immunotherapies.

## Data Availability

The original contributions presented in the study are included in the article/Supplementary Material, further inquiries can be directed to the corresponding author.
