# Supplementary material for: Identification of an Iron Metabolism-Related lncRNA Signature for Predicting Osteosarcoma Survival and Immune Landscape
Source: Front Genet. 2022 Mar 11;13:816460. doi: 10.3389/fgene.2022.816460 (PMC8961878; doi:10.3389/fgene.2022.816460)
Supplement: Supplementary file 1 [file Table1.docx]

**Supplementary Table 1:**

**Supplementary Table 1: 15 iron metabolism-related gene sets**

| **Gene sets name** | **Numbers of iron metabolism-related genes** |
| --- | --- |
| [GOBP_CELLULAR_IRON_ION_HOMEOSTASIS](http://www.gsea-msigdb.org/gsea/msigdb/geneset_page.jsp?geneSetName=GOBP_CELLULAR_IRON_ION_HOMEOSTASIS&keywords=IRON) | 70 |
| [GOBP_CELLULAR_RESPONSE_TO_IRON_ION](http://www.gsea-msigdb.org/gsea/msigdb/geneset_page.jsp?geneSetName=GOBP_CELLULAR_RESPONSE_TO_IRON_ION&keywords=IRON) | 9 |
| [GOBP_HEME_METABOLIC_PROCESS](http://www.gsea-msigdb.org/gsea/msigdb/geneset_page.jsp?geneSetName=GOBP_HEME_METABOLIC_PROCESS&keywords=IRON) | 33 |
| [GOBP_HEME_TRANSPORT](http://www.gsea-msigdb.org/gsea/msigdb/geneset_page.jsp?geneSetName=GOBP_HEME_TRANSPORT&keywords=IRON) | 10 |
| [GOBP_IRON_COORDINATION_ENTITY_TRANSPORT](http://www.gsea-msigdb.org/gsea/msigdb/geneset_page.jsp?geneSetName=GOBP_IRON_COORDINATION_ENTITY_TRANSPORT&keywords=IRON) | 14 |
| [GOBP_IRON_ION_HOMEOSTASIS](http://www.gsea-msigdb.org/gsea/msigdb/geneset_page.jsp?geneSetName=GOBP_IRON_ION_HOMEOSTASIS&keywords=IRON) | 86 |
| [MODULE_540](http://www.gsea-msigdb.org/gsea/msigdb/geneset_page.jsp?geneSetName=MODULE_540&keywords=IRON) | 10 |
| [REACTOME_IRON_UPTAKE_AND_TRANSPORT](http://www.gsea-msigdb.org/gsea/msigdb/geneset_page.jsp?geneSetName=REACTOME_IRON_UPTAKE_AND_TRANSPORT&keywords=IRON) | 58 |
| [GOMF_IRON_ION_BINDING](http://www.gsea-msigdb.org/gsea/msigdb/geneset_page.jsp?geneSetName=GOMF_IRON_ION_BINDING&keywords=IRON) | 150 |
| [HALLMARK_HEME_METABOLISM](http://www.gsea-msigdb.org/gsea/msigdb/geneset_page.jsp?geneSetName=HALLMARK_HEME_METABOLISM&keywords=IRON) | 200 |
| [HEME_BIOSYNTHETIC_PROCESS](http://www.gsea-msigdb.org/gsea/msigdb/geneset_page.jsp?geneSetName=HEME_BIOSYNTHETIC_PROCESS&keywords=IRON) | 100 |
| [GOMF_4_IRON_4_SULFUR_CLUSTER_BINDING](http://www.gsea-msigdb.org/gsea/msigdb/geneset_page.jsp?geneSetName=GOMF_4_IRON_4_SULFUR_CLUSTER_BINDING&keywords=IRON) | 42 |
| [GOMF_2_IRON_2_SULFUR_CLUSTER_BINDING](http://www.gsea-msigdb.org/gsea/msigdb/geneset_page.jsp?geneSetName=GOMF_2_IRON_2_SULFUR_CLUSTER_BINDING&keywords=IRON) | 22 |
| [GOBP_IRON_IMPORT_INTO_CELL](http://www.gsea-msigdb.org/gsea/msigdb/geneset_page.jsp?geneSetName=GOBP_IRON_IMPORT_INTO_CELL&keywords=IRON) | 11 |
| [GOBP_IRON_ION_TRANSPORT](http://www.gsea-msigdb.org/gsea/msigdb/geneset_page.jsp?geneSetName=GOBP_IRON_ION_TRANSPORT&keywords=IRON) | 79 |

**Supplementary figure legend:**

**Supplementary figure 1:** **Consensus clustering for 30 iron metabolism-related lncRNAs by the k-means method. (a)** Consensus matrices legend. **(b)** Delta area**. (c)** Consensus cumulative distribution function**. (d)** Tra**cking plot. (e-k)** consensus matrices for k = 3 - 10.

**Supplementary figure 2:** Differential expression of 30 prognosis-related lncRNAs. **(a-c)** Based on gender, age, and stage-specific, differential expression of 30 prognosis-related lncRNAs. **(d)** m2 macrophages enriched between Cluster 1 and Cluster 2.

**Supplementary figure 3: Evaluating the correlation between seven iron metabolism-related lncRNAs and overall survival of OS patients. (a-g)** Kaplan-Meier curves of seven iron metabolism-related lncRNAs. **(h)** ROC curve to evaluate 1-year prediction efficiency of other clinical fetures.
