# Supplementary figures and images for: Identification of an Iron Metabolism-Related lncRNA Signature for Predicting Osteosarcoma Survival and Immune Landscape
Source: Front Genet. 2022 Mar 11;13:816460. doi: 10.3389/fgene.2022.816460 (PMC8961878; doi:10.3389/fgene.2022.816460)

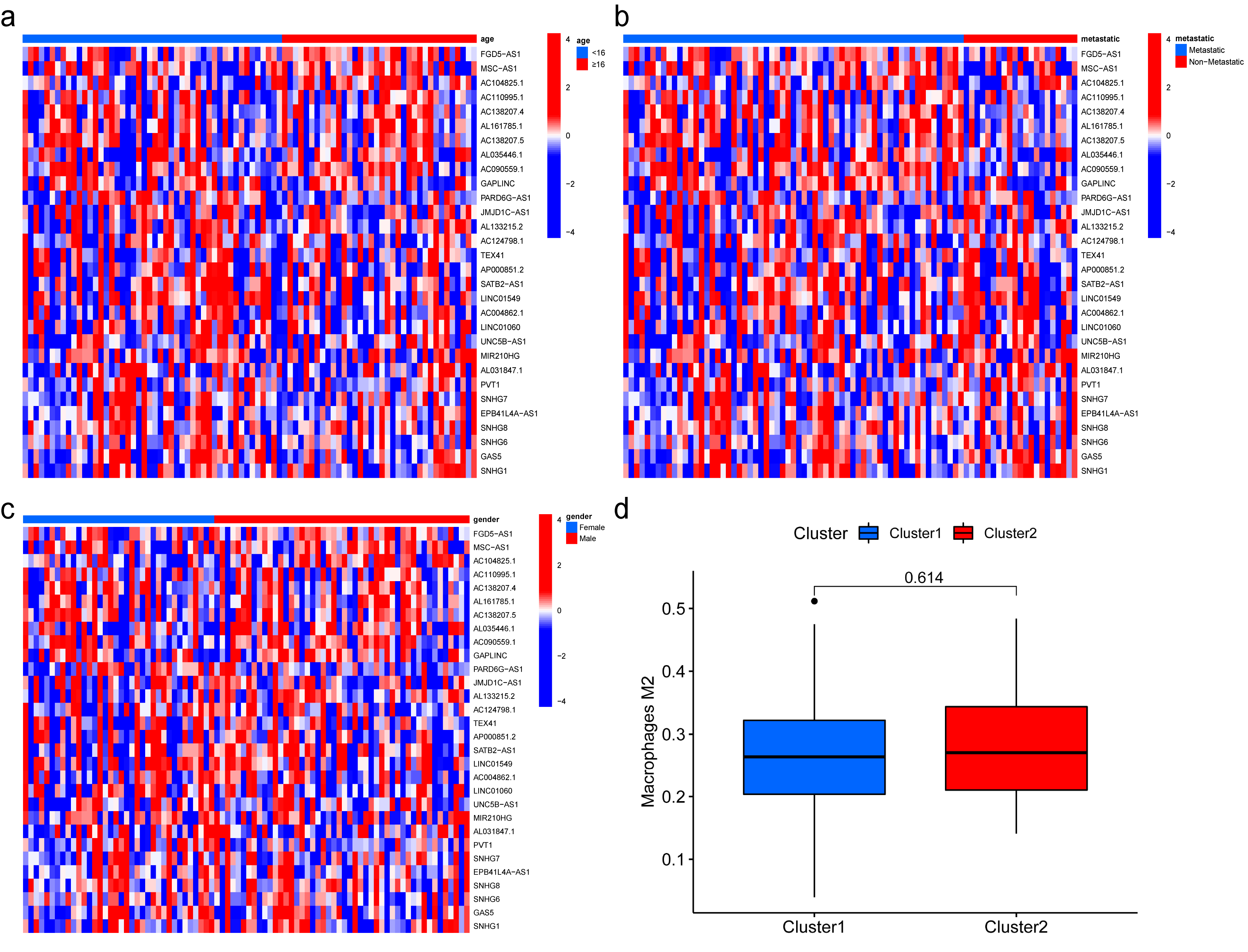

Supplement: Supplementary file 2 [file Figure8.TIF]

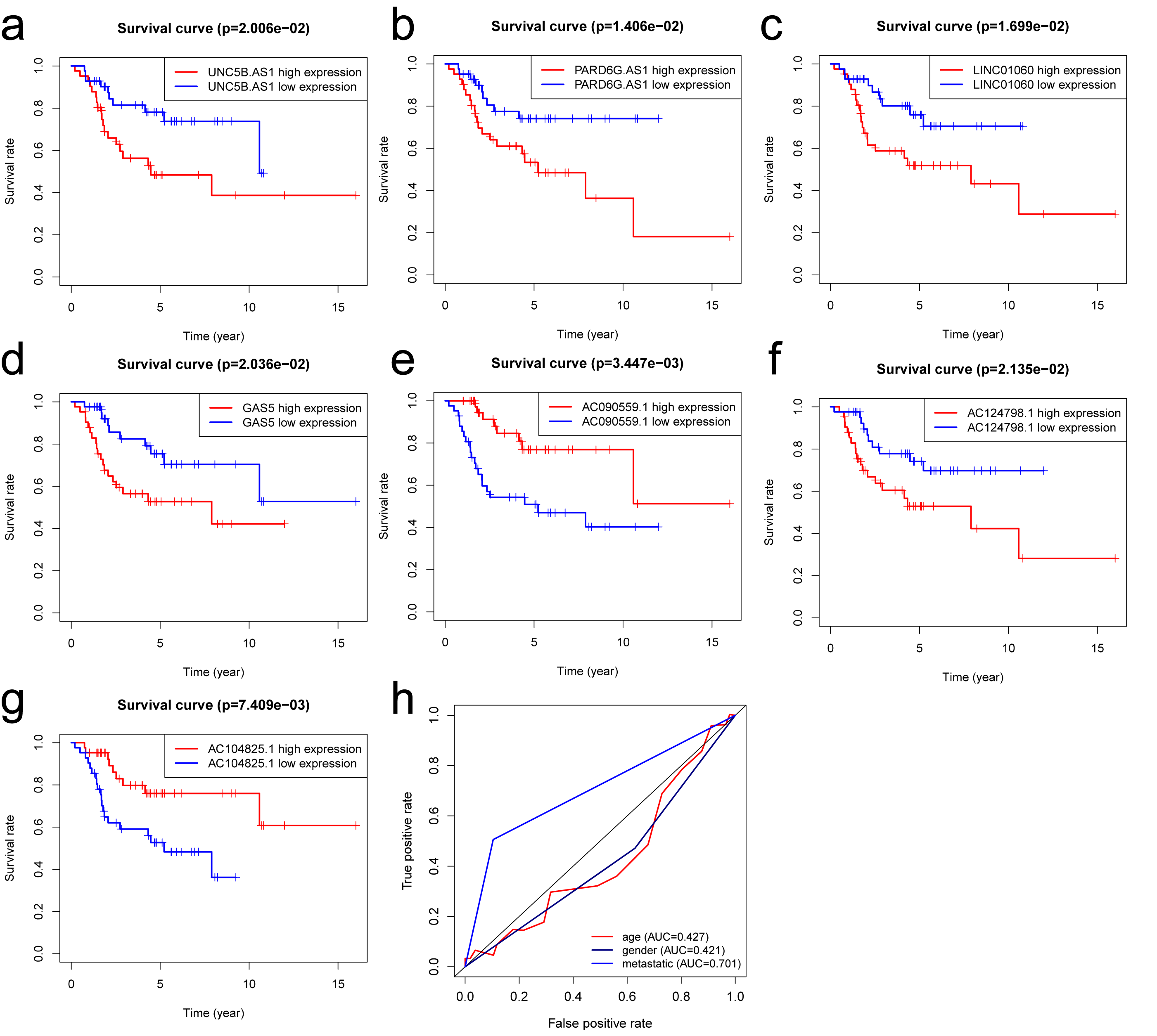

Supplement: Supplementary file 3 [file Figure9.TIF]

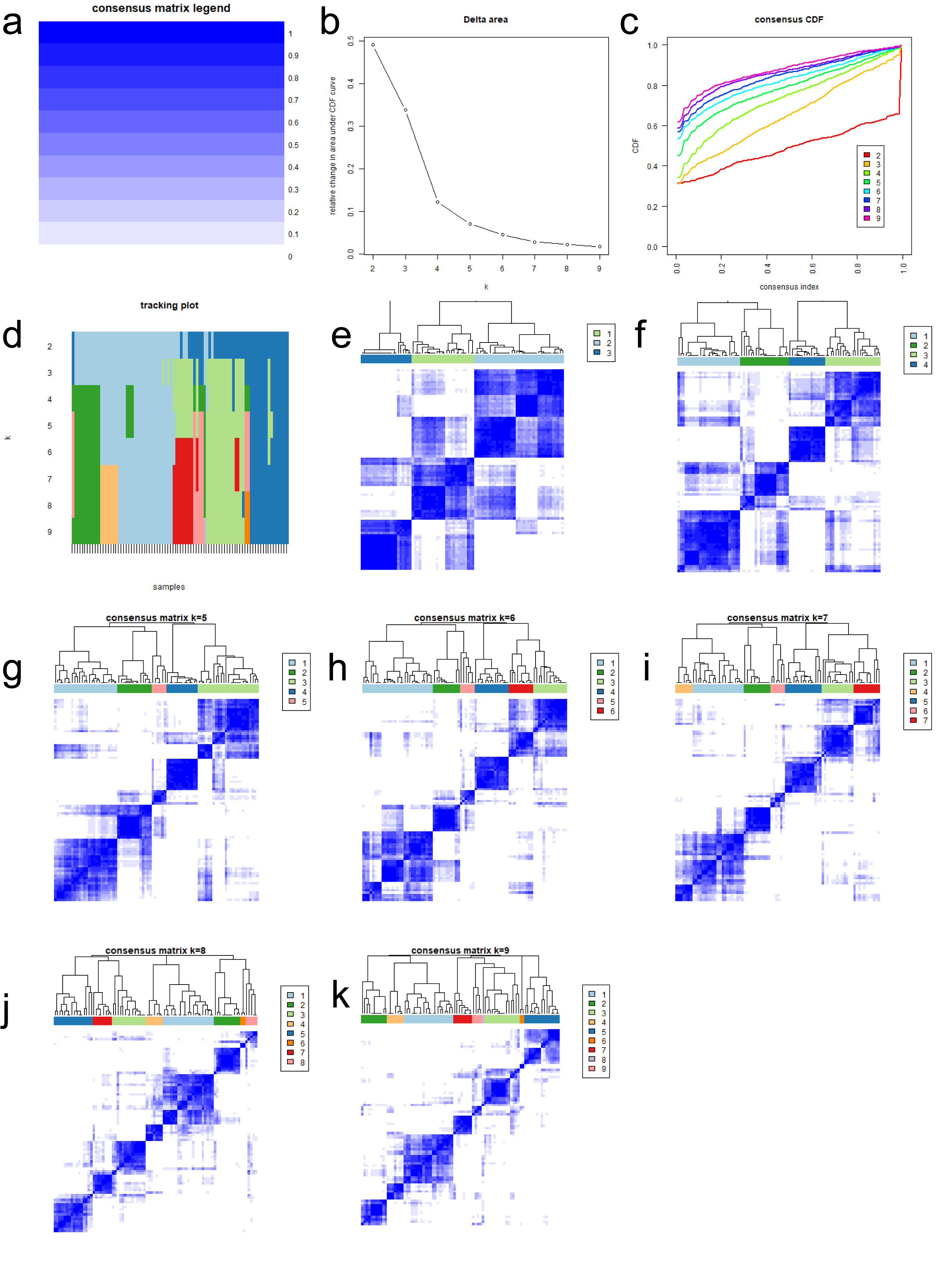

Supplement: Supplementary file 4 [file Figure7.TIF]
